# A phase II trial of chimeric monoclonal antibody G250 for advanced renal cell carcinoma patients

**DOI:** 10.1038/sj.bjc.6601617

**Published:** 2004-03-02

**Authors:** I Bleumer, A Knuth, E Oosterwijk, R Hofmann, Z Varga, C Lamers, W Kruit, S Melchior, C Mala, S Ullrich, P De Mulder, P F A Mulders, J Beck

**Affiliations:** 1 Department of Urology and Medical Oncology, University Medical Center, Geert Grooteplein 10, PO Box 9101, 6500 HB Nijmegen, The Netherlands; 2 Hospital Northwest, Frankfurt/Main, Germany; 3 Philipps-University, Marburg, Germany; 4 Daniel den Hoed Cancer Center, Erasmus MC, Rotterdam, The Netherlands; 5 Johannes Gutenberg University, Mainz, Germany; 6 Wilex AG, Munich, Germany

**Keywords:** monoclonal antibody, renal cell carcinoma, immunotherapy, WX-G250, CA-IX

## Abstract

Chimeric monoclonal antibody G250 (WX-G250) binds to a cell surface antigen found on >90% of renal cell carcinoma (RCC). A multicentre phase II study was performed to evaluate the safety and efficacy of WX-G250 in metastatic RCC (mRCC) patients. In all, 36 patients with mRCC were included. WX-G250 was given weekly by intravenous infusion for 12 weeks. Patients with stable disease (SD) or response were eligible to receive additional treatment for 8 weeks. None of the 36 enrolled patients experienced any drug-related grade III or IV toxicity. Only three patients had grade II toxicity possibly related to the study medication. In all, 10 patients had SD and received extended treatment. One complete response and a significant regression was observed during the follow-up of the treatment. Five patients with progressive disease at study entry were stable for more than 6 months after study entry. The median survival after treatment start was 15 months. The weekly schedule of WX-G250 was well tolerated. With a median survival of 15 months after the start of this treatment and two late clinical responses, WX-G250 seems to be able to modulate mRCC. To improve the activity of WX-G250-specific antibody-dependent cellular cytotoxicity and the clinical response rate, currently combinations of WX-G250 with cytokines are in phase II trials.

Patients with metastasised renal cell carcinoma (mRCC) have a poor prognosis. Up to one-third of the newly diagnosed patients present with metastatic disease and another 33% of the patients develop recurrent disease after surgery with curative intent (Mulders *et al*, 1997). In view of well-documented spontaneous remissions, the immune system appears to play a role in the natural history of the disease (Gleave *et al*, 1998). Against this background, the main focus of the treatment of mRCC has been on immunotherapy. Currently, interferon-*α* (IFN-*α*) and interleukin-2 (IL-2) are most commonly used in the treatment of mRCC, either alone or in combination (Negrier *et al*, 1998; Medical Research Counsil, 1999). However, the response rates are low and the toxicity significant. Therefore, new treatment modalities are being studied (Nathan and Eisen, 2002; Oosterwijk-Wakka *et al*, 2002).

Currently, several monoclonal antibodies (mAbs) have been approved for the use of cancer treatment, for example, in non-Hodgkin's lymphoma (McLaughlin *et al*, 1998). In RCC, the chimeric monoclonal antibody WX-G250 has been identified and developed for both diagnostic and therapeutic purposes (Oosterwijk *et al*, 1986; Steffens *et al*, 1997). It recognises the CA-IX^MN/G250^ antigen expressed in >95% of RCC of the clear cell type (Grabmaier *et al*, 2000). Moreover, CA-IX^MN/G250^ is not expressed in normal kidney tissue and in other normal tissues, the expression is highly restricted and limited to large bile ducts and gastric epithelium (Oosterwijk *et al*, 1986). The observation that WX-G250 was able to mediate antibody-dependent cellular cytotoxicity (ADCC) against several RCC cell lines (Surfus *et al*, 1996) led to the initiation of the present study.

## PATIENTS AND METHODS

### Study design

A phase II, prospective, open-label, single-arm, multicentre study was initiated. Patients with mRCC received weekly doses of 50 mg WX-G250 for 12 weeks. Study objectives included the evaluation of efficacy, safety, immunogenicity and biological activity of the WX-G250. Patients were treated at five medical centres: Johannes-Gutenberg University Hospital Mainz, Germany (enrolled patients: *n*=14); Hospital Northwest Frankfurt/Main, Germany (*n*=11); University Medical Center Nijmegen, The Netherlands (*n*=6); Philipps University Hospital, Marburg, Germany (*n*=4); and Daniel den Hoed Cancer Center, Erasmus MC, Rotterdam, The Netherlands (*n*=1). All patients signed an informed consent approved by the local ethical committees of the respective hospitals. Patients were recruited between 14 June 2000 and 20 December 2000.

### Patient population

All patients had primary RCC of clear cell histology and prior nephrectomy. The inclusion criteria were: bidimensionally measurable disease with lesions <5 cm in diameter and at least one lesion of ⩾1 cm; life expectancy >28 weeks; Karnofsky performance status ⩾70%; seronegative for human immunodeficiency virus and hepatitis B surface antigen; absolute neutrophil count ⩾2.0 × 10^8^/dl; platelet count ⩾100 × 10^8^/dl; haemoglobin >6.5 mmol/l (equals 10.5 g/dl); total bilirubin <1.5 × upper limit of normal (ULN); AST, ALT <3 × ULN (<5 × ULN if liver metastases is present). Patients with clinical signs of CNS metastases and patients with bone metastases only were excluded.

### Antibody

WX-G250 is a human/mouse chimeric mAb (IgG1 isotype) derived from murine mAb G250 by recombinant DNA techniques. WX-G250 is similar with regard to binding affinity and specificity in comparison with the murine G250 IgG (Steffens *et al*, 1997). All clinical lots were generated starting from a cell bank according to current GMP requirements. The antibody was supplied by Wilex AG (München, Germany).

### Treatment

Patients received 50 mg WX-G250 once a week by intravenous infusion for 12 weeks followed by radiographic evaluation 4 weeks later. Patients with stable disease (SD) or a tumour response were eligible for extended treatment, consisting of eight additional weekly infusions of WX-G250. The WX-G250 was dissolved in 10 ml solution for injection, prefiltered through a low-protein binding 0.2 *μ*m filter and infused in 50–100 ml normal saline over a period of 30 min. Patients were observed for at least 2 h for blood pressure, pulse, temperature and allergic reactions after completion of the infusion. The 50 mg dose was chosen based on the following observations: (1) a single dose of 50 mg WX-G250 (radiolabelled with ^131^Iodine) was safe in 16 RCC patients (Steffens *et al*, 1997); (2) 50 mg WX-G250 was sufficient to reach maximum uptake of the antibody in RCC (Steffens *et al*, 1997); and (3) the results of a multidose phase I study with escalating WX-G250 doses showed that weekly doses of 25 mg/m^2^ (corresponding to the 50 mg dose used in this study) caused no clinical or laboratory drug-related adverse events during 6 weeks treatment (Wiseman *et al*, 2001). The interval of the application was selected based on the WX-G250 serum half-life of approximately 70 h (Steffens *et al*, 1997). Therefore, it was expected that the levels of WX-G250 adequate to induce ADCC would persist for at least 1 week (Surfus *et al*, 1996). Weekly administration was not expected to lead to significant cumulative increase of serum levels of WX-G250.

### Human anti-chimeric antibody (HACA) evaluation

A sandwich-type ELISA was used to analyse HACA levels in the serum of patients as described previously (Steffens *et al*, 1997). In brief, unconjugated WX-G250 was coated onto ELISA plates, followed by incubation with the serum of the patient. The detection of anti-WX-G250 HACA was performed using biotinylated WX-G250, and a streptavidin-biotinylated peroxidase complex. In 22 patients, pretreatment HACA levels were determined. Blood samples for HACA evaluation were taken before each infusion with WX-G250 and regularly during follow-up. The serum of a patient with positive HACA levels obtained from an unrelated WX-G250 trial was used as positive control. The calibration curve for the quantification of anti-WX-G250 HACA was generated using the WX-G250 anti-idiotypic antibody NUH82 (Uemura *et al*, 1994). The limit of detection (LOD) of this ELISA was 8.3 ng/ml (NUH82); the limit of quantification (LOQ) was 27 ng ml^−1^.

### Biological activity, ADCC

At baseline and prior to WX-G250 infusion at weeks 4 and 12, venous blood samples were collected in four sodium heparin *vacutainer*™ CPT™ tubes (Becton, Dickinson and Company, The Netherlands). The tubes were centrifuged (1500 **g**, 30 min) at the treatment site and shipped overnight to the evaluator. Peripheral blood mononuclear cells (PBMCs) were collected and frozen in 50% FCS/10% DMSO medium and stored at −170°C until the time of evaluation. To confirm the presence and proportion of natural killer (NK) lymphocytes, immunophenotyping was performed. The biological activity of WX-G250 was analysed by 4-h ^51^chromium release assays at different effector to target cell ratios using autologous cryopreserved PBMC as effector cells. Target cells were SKRC-17pMW1-c14 (CA-IX^MN/G250^ antigen-transfected RCC cell line) and SKRC-17 (CA-IX^MN/G250^ antigen-negative RCC cell line). As controls for lytic activity of the PBMC, P815 cells were used as target cells in the presence of anti-P815 rabbit antiserum (‘classic’-ADCC). To evaluate the nonspecific cellular lytic activity, K562 and Daudi cells were used as additional target cells, in the absence of WX-G250 antibody. The cytolytic activities were expressed as percentage weighted mean of specific cytolysis (Lamers *et al*, 1991). Antibody-dependent cellular cytotoxicity measured against the target cells incubated with the WX-G250 antibody (1 *μ*g ml^−1^) was corrected for the ADCC seen against the target cells incubated without the WX-G250 antibody.

### Patient monitoring and efficacy evaluation

Patients were monitored for safety, biological activity of WX-G250, HACA development and clinical antitumour effects. At baseline and weeks 4, 12 and 16 medical histories, physical examinations, urinalysis and laboratory studies including complete blood count and chemistry panel were performed. Toxicity was evaluated according to the Common Toxicity Criteria (Version 2.0, April 1999, National Cancer Institute). Computed tomography (CT) scans of the thorax and abdomen were performed at baseline, at week 16 and 4 weeks after the extended treatment, when applicable. Tumour responses were evaluated according to the following WHO criteria: (1) *Complete response* (CR): the disappearance of all known disease determined by two evaluations not less than 4 weeks apart. (2) *Partial response* (PR): ⩾50% decrease in the sum of products of largest and perpendicular diameters of the lesions that have been measured to determine the effect of therapy by two evaluations not less than 4 weeks apart, in the absence of new lesions or progression of any lesion. (3) *SD*: the total tumour size has less than 50% and the increase is less than 25% in the size of one or more measurable lesions. (4) *Progressing disease* (PD): a 25% or more increase in the size of one or more measurable lesions, or the appearance of new lesions. For the CT and MRI evaluations an independent central review was performed as requested by protocol.

### Statistical methods

The study had a two-stage design. In the first stage, 32 evaluable patients with mRCC were included. In all, 22 additional patients were allowed to participate (54 patients in total) if at least three objective responses were observed at the time of the radiological evaluation at week 16. The study had to be terminated if less than three patients or more than five had an objective response. At the maximum enrolment number of 54 patients, the trial was powered at 80% and based on *α*⩽0.05 to detect a difference between an assumed spontaneous response rate of 5% and an underlying true response rate of 15%.

## RESULTS

### Patient characteristics (Table 1)

**Table 1:** Characteristics of the evaluated patients

**Pt. no.**	**Sex**	**Age (years)**	**Prior tumour therapy (best response)**	**Karnofski (%)**	**Clinical status at start**	**Metastasis**	**Number of infusions**	**Response after first cycle**	**Survival (months; until January 2002)**
1	M	68	IFN+Vinbl (PD)	90–100	PD	Adrenal	12	PD	6
2	F	45	IFN+Vinbl (PD)	70	PD	Lung and liver	4	PD	2
3	F	75	None	80	PD	Lung	12	PD	4
4	M	51	None	80	PD	Lung, liver and LN	11	PD	3
5	F	77	None	90	PD	Lung	5	PD	2
6^a^	M	66	IFN+Vinbl (SD)	80	PD	Lung and liver	20	SD	18+
			IFN+Vinbl (PD)						
			5FU+CF (PD)						
7	F	58	Radiation (PD)	90–100	PD	Lung	12	PD	18+
8	M	77	IFN+Vinbl	80	PD	Lung and bone	4	PD	Date unknown
			5FU+CF						
9^a^	M	68	IFN+Vinbl (SD)	90–100	PD	Lung, pleura, adrenal	20	SD	18+
			5FU+CF (SD)						
10	M	69	IL-2/IFN/5FU (SD)	90–100	PD	Lung and liver	12	PD	18+
			IL-2/IFN/5FU red (PD)						
11	M	66	None	90–100	PD	Lung and LN	12	PD	17
12	F	67	None	90–100	UN	Liver, spleen and LN	12	PD	18+
13	F	64	IFN+Vinbl (PD)	90–100	PD	Lung and liver	11	PD	4
14	M	72	None	90–100	PD	Lung	7	PD	1
15	M	51	IFN+Vinbl (PD)	90–100	PD	Lung, LN, pancreas	12	PD	17+
16	M	54	IFN+Vinbl (PD)	90–100	PD	LN, contralateral kidney and psoas muscle	12	SD	17+
			IFN+*cis-*RA (PD)						
			5FU+CF (PD)						
17	M	67	IFN+Vinbl	80	PD	Lung	5	PD	7
18	M	50	IFN (PD)	90–100	PD	Lung	12	PD	9
19	M	43	None	90–100	PD	Lung and LN	12	PD	8
20	F	60	None	80	UN	LN	12	PD	11
21^a^	M	69	None	90–100	UN	Lung and LN	20	SD	16+
22^a^	M	70	None	90–100	UN	LN	20	SD	13
23^a^	M	61	None	80	PD	Lung, LN and Liver	20	SD	16+
24^a^	M	56	IL-2 (SD)	90–100	PD	LN	20	SD	15+
			DC vaccination (SD)						
25^a^	F	52	IFN+*cis-*RA (CR)	90–100	PD	Other kidney	20	SD	15+
26	M	69	IL-2/IFN/5FU (PD)	80	PD	Lung and LN	12	PD	15+
27^a^	M	57	IL-2/IFN/5FU (PR)	90–100	UN	Lung	20	SD	15+
28^a^	F	76	None	90–100	PD	Lung and adrenal	20	SD	14
29	M	66	IL-2/IFN/5FU (SD)	80	PD	Lung and LN	12	PD	15+
			IL-2 inhalation (PD)						
30	F	65	IL-2/IFN/5FU (PD)	90–100	PD	Lung and LN	12	PD	14+
			Toremifene+Vinbl (PD)						
31	F	70	IFN+Vinbl (PR)	90–100	PD	Lung	12	PD	14+
			IFN+Vinbl (PD)						
			IFN+Vinbl (PD)						
32	M	76	None	90–100	PD	Lung	12	PD	14+
33	M	71	None	80	PD	LN and pleura	12	PD	14+
34	M	57	Vaccination (PD)	90–100	PD	Lung	12	PD	7
35	M	67	IFN+*cis-*RA (SD)	90–100	PD	Lung and LN	10	PD	5
36^a^	F	73	None	80	PD	Lung	20	SD	13+

a Patients who received extended treatment.

*Sex*: M=male, F=female; *response*: PD=progressive disease, SD=stable disease, PR=partial response, CR=complete response, UN=unknown; *metastasis*: LN=lymphnode; *prior treatment*: IFN=interferon-alpha, Vinbl=vinblastine, 5FU=5-fluorouracil, CF=calcium folinate, IL-2=interleukin-2, *cis-*RA=*cis*-retinoic acid, DC=dendritic cell.

From June 2000 to December 2000, a total of 36 RCC patients were included. The study population (mean age 64 years, range: 42–77 years, 12 females and 24 male) displayed a total of 107 target lesions in different sites at study entry. In 29 patients (80.5%), disease progression was documented before study entry, for four patients the status was not rated, the remaining three were not progressive. Of the 36 evaluated patients, 20 patients received prior systemic treatment that resulted in one CR, one PR, three SD. In all, 15 patients did not respond to cytokine treatment.

### Safety evaluation (Table 2)

**Table 2:** Adverse events registered during the study, stratified by NCI class and grade

**NCI class**	**Events (*N*)**	**Pts (*N*)**	**Pts (%)**	**n.a.**	**Gr I**	**Gr II**	**Gr III**	**Gr IV**
Pain	32	15	41.7	0	12	13	7	0
Gastrointestinal	26	14	38.9	0	14	12	0	0
Pulmonary	20	9	25.0	0	5	5	6	4
Cardiovascular (general)	17	12	33.3	0	8	4	5	0
Constitutional symptoms	15	9	25.0	0	10	4	1	0
Neurology	11	8	22.2	0	3	6	2	0
Blood/bone marrow	10	5	13.9	0	1	5	4	0
Not to classify	7	5	13.9	4	3	0	0	0
Renal/genitourinary	5	4	11.1	0	2	1	2	0
Haemorrhage	5	3	8.3	0	3	0	1	1
Hepatic	4	2	5.6	0	0	0	4	0
Infection/febrile neutropenia	3	3	8.3	1	1	1	0	0
Metabolic/laboratory	3	3	8.3	0	1	1	1	0
Allergy/immunology	1	1	2.8	0	0	1	0	0
Ocular/visual	1	1	2.8	0	0	1	0	0

Total	160	30	83.3	5	63	54	33	5

Pts=patients; Gr=grade; n.a.=grade not specified.

All 36 patients received at least one dose of WX-G250 and were assessed for safety. No dose reduction was necessary. Of the recruited 36 patients, 30 experienced a total of 160 adverse events during the course of the study. All grade 3 and 4 toxicities were considered not to be related to the study medication. An overview of the observed toxicities is given in Table 2. The grade 4 toxicities consisted of dyspnoea and pulmonary insufficiency (patient 4), dyspnoea at rest (3), respiratory insufficiency (5) and gastrointestinal bleeding (13). Two patients (4,5) died during the study (defined as death within 30 days after the last application of study medication), both due to progression of their disease. None of the serious adverse events (including the deaths) were related to study medication, but due to underlying disease.

### Evaluation of HACA

In the evaluated pretreatment sera of 22 patients, no HACA levels were detected. In three samples of two patients (18,27) levels higher than the LOD could reproducibly be detected. Serum of patient 18 showed an equivalent of 76 ng ml^−1^ anti-G250 antibody NUH82 at treatment week 12. In the sera of patient 27, an equivalent of 49 ng ml^−1^ anti-G250 antibody NUH82 at extension week 1 and a value between the LOD and LOQ at extension week 8 could be detected.

### Immunological monitoring (Table 3)

**Table 3:** Immunophenotyping and ADCC

**Subject**	**Treatment day**	**Immunophenotyping % NK cells**	**Cytolytic activity cG250-ADCC**	**Cytolytic activity ‘classic’ ADCC**	**NK activity**
#01	0, 21, 76	5, 4, 4	16, 9, 10	40, 29, 27	−, 5, 4
#06	0, 21, 77	9, 9, 7	14, 14, 11	38, 32, 40	16, 19,−
#07	0, 21, 77	9, 11, 9	8, 16, 5	94, 100, 50	−, 35, −
#09	0, 28, 77	7, 8, 11	1, 1, 2	45, 72, 49	−, 24, 28
#10	0, 21, 77	6, 5, 2	0, 1, 0	40, 32, 38	−, −, −
#11	0, 21, 77	8, 8, 5	10, 7, 5	55, 42, 48	−, −, −
#12	0, 21, 77	ND	3, 3, 2	32, 39, 36	−, −, −
#13	0, 21, 78	ND	2, 2, 2	27, 27, 22	−, −, −
#15	0, 21, 77	ND	3, 1, 1	20, 16, 21	−, −, −
#16	0, 21, 78	14, 13, 10	14, 25, 26	37, 47, 50	4, 8, 6
#18	0, 29, 85	1, 1, 1	0, 2, 0	49, 37, 15	0, 1, 0
#19	0, 21, 77	6, 2, 1	1, 0, 0	34, 38, 9	0, 0, 0
#20	0, 21, 77	1, 1, 2	1, 2, 3	2, 6, 8	0, 2, 1
#21	0, 21, 77	12, 12, 7	4, 4, 3	25, 23, 17	5, 5, 3
#22	−7, 21, 77	7, 4, 5	15, 7, −	31, 40, −	29, 16, −
#24	−8, 21, 77	9, 4, 4	6, 5, 5	55, 36, 41	17, 16, 45
#25	0, 21, 77	7, 7, 5	14, 15, 18	32, 37, 36	24, 23, 38
#26	0, 50, 78	6, 4, 13	0, 0, 3	16, 20, 40	3, 6, 20
#27	0, 21, 77	5, 4, 5	7, 9, 11	44, 32, 32	−, −, −
#28	0, 28, 84	2, 3, 3	0, 0, 0	24, 22, 19	4, 4, 4
#29	0, 28, 78	1, 1, 2	0, 1, 2	9, 8, 17	0, 0, 1

NK=natural killer cell; ADCC=antibody-dependent cellular cytotoxicity; ND=no data; %NK cells=percentage NK cells of viable leucocytes. Cytolytic activities expressed as %WMSL at effector-to-target-ratio of 20 : 1.cG250-ADCC : cG250 mAb-induced cytolysis of G250-ligand-expressing target cell. ‘classic’-ADCC: anti-P815 Ab-induced cytolysis of P815 target cell (=positive control).

NK activity: cytolysis of NK-sensitive target cell K562.

In 18 patients phenotypic analysis of PBMC was performed (Table 3). Consecutive samples of every patient were tested simultaneously to exclude assay-to-assay variation. In 15 of these 18 patients, the percentage of NK cells was >2% of leucocytes in at least one of the samples (range: 1–14%). The percentage NK cells in individual patients remained rather constant during treatment. Consecutive samples of 21 patients were tested for ADCC capacity (Table 3). Nine out of 21 patients showed moderate (5–25%) to high (>25%) levels of WX-G250-ADCC activity. Five out of 21 patients showed low levels of WX-G250-ADCC and seven out of 21 patients showed no WX-G250-ADCC activity. Of the 21 patients, 19 tested showed normal levels of ‘classic’-ADCC activity. In general, WX-G250-related ADCC remained unchanged during the treatment.

### Clinical response (Table 1)

Clinical progression before week 16 was seen in eight patients. In four of these patients progression occurred within 6 weeks after study entry and further patients were recruited as replacements, in accordance with the protocol. Patient 16 was judged as SD according to an outside radiologist, but was subsequently lost to follow-up. Independent central tumour assessment was performed at week 16 in the remaining 27 patients. Radiographically proven progression of the disease was observed in 17 patients, who therefore did not qualify for extended treatment with WX-G250. Of these, 10 patients showed SD after 12 weeks of treatment and continued treatment consisting of eight additional weekly infusions of WX-G250. The clinical status of these patients was assessed in week 24 and eight out of 10 patients still showed SD. After completion of the treatment patients were routinely followed at 3 monthly intervals. The follow-up evaluation revealed a minor response in patient 9 in week 44 and a CR in patient 21 in week 38. The evaluation of survival showed a median time to death of 15 months after the start of the WX-G250 treatment. The evaluation of the first stage of the study did not show three objective responses at the time of radiological evaluation at week 16. Consequently, the study did not enter the second stage of the trial.

## DISCUSSION

We performed a phase II, prospective, open-label, single-arm, multicentre study in patients with advanced RCC receiving weekly doses of 50 mg WX-G250 for 12 weeks to evaluate the potential therapeutic effect of WX-G250. WX-G250 recognises the antigen CA-IX^MN/G250^ that is homogeneously expressed in virtually all clear cell RCC. More importantly, the expression in normal tissue is extremely limited, making this antigen an attractive target for immunotherapy.

During the trial no serious drug-related clinical or laboratory adverse events were observed, and no dose adjustment was necessary. Therefore, the toxicity profile of WX-G250 is favourable and comparable with prior studies evaluating WX-G250 (Steffens *et al*, 1997). WX-G250 is a murine-human chimeric antibody, and may consequently induce the development of HACA. In the current trial, low levels of HACA were detected in only two out of 35 patients (eight and 27). The HACA levels were not associated with clinical symptoms, which is in accordance with earlier studies evaluating WX-G250 (Steffens *et al*, 1997). This study demonstrates that multiple infusions with WX-G250 are well tolerated and can be given safely.

The evaluation of the first stage of the trial showed less than three objective responses needed to continue into the second stage of the trial and thus the trial was terminated. Nevertheless, the follow-up revealed two objective responses: patient 9 presented at study entry with multiple progressive metastases. After both the first course and extended treatment, the lesions were rated as stable. In the following months, radiological evaluation showed a decrease of the target lesions. Taking all measurable lesions into account, the response remained slightly below the 50% decrease required for a partial remission, making it a minor response. Patient 21 started treatment with WX-G250 immediately after nephrectomy. At study entry he presented with lymph node metastases and multiple pulmonary lesions. The size of the lesions decreased 29% between weeks 16 and 24, and was therefore rated as SD. In week 38 after study start, a complete remission of all the lesions was documented. Since spontaneous remissions of RCC metastases after the removal of the primary tumour do occur (Gleave *et al*, 1998), we cannot exclude that the clinical observation of patient 21 was a reflection of the natural history of the disease, unrelated to WX-G250.

In all, 11 patients were rated with SD at the radiological evaluation at week 16. Of those patients, eight showed PD at study entry, of which two patients (6,23) with previously progressive liver lesions and others after progression under previous immunotherapy regimens. These are events that rarely occur, even after ‘standard’ cytokine treatment (Lissoni *et al*, 1994; Escudier *et al*, 1999; Motzer *et al*, 2002). Collectively, these results suggest that WX-G250 has the capacity of modulating the natural history of metastasised RCC with a safe toxicity profile.

Antibody-dependent cellular cytotoxicity is suggested to be the main effector mechanism of WX-G250 (Surfus *et al*, 1996) and is mediated by the interaction between the Fc region of an antibody bound to a tumour cell and the Fc*γ* receptors on immune effector cells, such as neutrophils, macrophages and NK cells (Clynes *et al*, 2000). In our trial, the levels of WX-G250-mediated ADCC differed between the patients: 42% of the patients showed moderate ADCC, whereas 33% showed no ADCC at all. There was no clear correlation between the proportion of NK cells and the level of WX-G250-mediated ADCC and no correlation between the *in vitro* levels cytotoxicity and the clinical responses. This high variability in observed ADCC capacity and number of NK cells was also found in healthy donors (personal observations), suggesting that the observed variation was not the result of the disease status of the patients. Molecular studies have shown significant polymorphism in the genes for the different Fc receptors (Vance *et al*, 1993). These polymorphisms may have important functional consequences. The pattern of Fc*γ* RIIIA expression polymorphism is probably correlated with the ability of NK cells to perform ADCC. This may be the reason that no correlation between the proportion of NK cells and the level of WX-G250-mediated ADCC was observed.

In summary, the weekly schedule of intravenous WX-G250 in patients with mRCC was safe and well tolerated. The evaluation of the immunogenicity of WX-G250 demonstrated that an increased level of HACA does not lead to clinical symptoms. In our trial, one complete responder, one minor response and a substantial number of durable disease stabilisations were observed with WX-G250 monotherapy. The median survival after study entry was 15 months. This suggests the capacity of WX-G250 to modulate the natural history of metastatic RCC. Recently, it was shown that a variety of cytokines, for example, IL-2 and IFN*γ*, led to the upregulation of WX-G250-mediated ADCC (Liu *et al*, 2002). Subsequently, phase II trials optimising treatment schedules with WX-G250 by combination with cytokines have been initiated.
